# Does randomization matter in rare disease clinical trials

**DOI:** 10.1371/journal.pone.0339427

**Published:** 2026-09-02

**Authors:** Stephanie Wied, Denis Kirilov Razsolkov, Nicole Heussen, Ralf-Dieter Hilgers

**Affiliations:** 1 Institute of Medical Statistics, RWTH Aachen University, Aachen, Germany; 2 Medical Faculty, Sigmund Freud Private University, Vienna, Austria; University Hospital Cologne: Uniklinik Koln, GERMANY

## Abstract

**Background:**

Conducting Rare Disease Clinical Trials poses a wide variety of research challenges. Many techniques that are usually considered the best standard for evaluating new therapies are more difficult to implement here. This also holds in terms of randomization in clinical trials. The question arises how to randomize and, especially, which randomization procedure (RP) should be used to prevent the influence of bias focussing on allocation bias as efficiently as possible.

**Methods:**

We present guidance that supports users in the selection and implementation of an RP based on the ERDO framework, which is a template for the evaluation of RPs for design optimization. For this purpose, we use the freely available R-package randomizeR, which is an easy-to-use tool to evaluate different RPs for various study designs while quantifying different types of bias. The evaluation is based on different metrics, such as the mean type-1 error probability or the go-no-go criterion, and brings these together with indirect evaluation criteria such as predictability and the number of correct guesses.

**Results:**

For the evaluation of RPs and the comparison of RPs with each other, both the presented indirect and direct criteria should be considered together. Examining the criteria individually might be misleading and may lead to different decisions regarding the RP. Different study designs may result in different assessments, so there is no universal solution or choice for RPs.

**Conclusion:**

Randomization is and will continue to be a key methodology for rare disease clinical trials. Quantification of bias in particular with respect to the test decision is an essential issue, and the use of the ERDO framework should be a routine practice in the design of a trial.

## 1 Background

Rare Diseases (RD) is an area where national and international multidisciplinary collaborations are valuable to overcome some of the present challenges in research. Comparative trials serve as a major basis for evaluating new therapies in RD patient care. Randomization, as a tool for treatment allocation in comparative trials, and blinding are the accepted standards for enabling a fair treatment comparison by mitigating bias. The unique challenges of RD clinical trials originate from the limited sample size, related, for instance, to heterogeneity in the patient population, so that researchers may think about restricting or totally avoiding randomization. In [1], it was stated that properties of randomization procedures relying on approximate arguments fail to suffice in small clinical trials. Recent findings underpin the view that randomization in small population clinical trials, like in RD, is different from classical clinical trials [2]. The above-mentioned challenges, such as small sample sizes caused by various factors, such as geographically widely distributed cases, limited patient populations, and ethical constraints, all affect the design, implementation, and interpretation in terms of evidence of an RD clinical trial. In particular, randomization is affected directly by these challenges, as, e.g., in small sample sizes the risk of imbalanced group sizes is increased, resulting in the need to use methods to mitigate imbalance or incorporate stratification approaches [3]. Furthermore, as specific reporting recommendations on the applied randomization technique details are missing it result incomplete reporting of methodological aspects and a lack of transparency on bias risks and their management [4]. In particular, the choice of the randomization procedure should be explicitly justified, analogous to the justification required for sample size planning, which is currently not included in guidelines and specific guidance on how this justification should be reported are missing. This also results in insufficient understanding of randomization methods in RD clinical trials, which leads to uncertainties in study design [5]. Regardless of the difference in the value of randomization, in RD clinical trials, more attention should be paid to the selection of the best-performing randomization procedure. This includes, on the one hand, improved mitigation of bias, and on the other hand, the potential for an alternative basis for the inference [6].

Correspondingly, the first [7] of a series of webinars [8,9] launched by the European Joint Program on Rare Disease was dedicated to randomization in RD clinical trials. The 148 subscribers of this webinar included participants around the world, including 30 from the United States. They are from almost all professional areas dealing with clinical trials, i.e., health care professionals (n = 57), methodologist (n = 53), or from industry (n = 51). There is a great interest from the European Reference networks in learning about randomization issues (n = 51 participants). The major reasons to use randomization in RD clinical trials are to increase the validity of the trial (64%) and to fulfill regulatory issues (27%). There is some doubt that randomization can be used because the number of patients is too small. Furthermore, the complexity of the disease and trials mitigate the use of randomization. In general, the patients refuse to participate in such trials because of the chance to be allocated to the control group. In particular, the restricted sample size is elaborated in the webinar with respect to finding the best procedure. The selection process of the randomization procedure gives some indication about expert knowledge. In most of the cases (41%), the selection was based on advice from the institutional clinical research department with a dedicated statistician, on the standard requirement of the pharmacompany/CRO (24%), or on personal expertise (23%). Remarkably, it is stated that many initiatives/publications guide what kind of randomization to choose, including some simulated impacts of choosing one randomization method over another. Although this requires careful input from statistical experts, a user-friendly and openly accessible tool with a dynamic display (visualization) of the impact of choosing one randomization method over another, with examples of successive randomization implementation, may be needed.

Randomization is intended to help obtain unbiased estimators in treatment comparisons. In particular, this aims to mitigate allocation bias arising from the investigator’s potential influence on the allocation of patients to treatment groups [10]. Furthermore, it encourages balance between treatment arms with respect to both known and unknown confounders. Thus, it plays an important role in the design, conduct, and analysis of the clinical trial [6]. In the case of weak concealment of the randomization sequence, the investigator may know or predict which intervention the next eligible participant should receive. This may influence the investigators decision to enroll a potentially eligible participant to the trial such that participants with a good prognosis (i.e., expected good outcomes and response to treatment) to be assigned to one group rather than another, i.e., it result in allocation bias. Concealment of allocation process can be used for prevention, which includes the information on details of the randomization process such as block sizes. Similar, study participants who are assigned to an intervention earlier may respond differently from those participants who are recruited later which result in chronological bias. As a preventive measure, the use of small block sizes may be helpful, but must be carefully evaluated against the resulting risk of allocation bias [11].

This paper aims to guide and support investigators in selecting the best Randomization Procedure (RP) for different trial settings while accounting for the assessment of potential biases. It demonstrates how the open-access R-package randomizeR [12] can be used to support both the selection of a suitable RP and the preparation of a corresponding randomization list.

The paper is organized as follows. Section 2 is intended for setting up the scene while providing an overview on randomization and RPs, followed by our review of currently used randomization and blinding practices in RD trials, presenting the methodology for evaluating bias and demonstrate that the impact of allocation bias becomes more pronounced in small samples. We further discuss key properties of RPs and present an overview of existing methods for different study designs, and a description of our simulation study. Section 3 presents the results of the review and the simulation studies, followed by the conclusion in Section 4.

## 2 Methods

### 2.1 Randomization schemes

In a recent New England Journal of Medicine paper, Collins [13] clarified that the term “randomization” refers to a method for treatment allocation but does not refer to a methodology, which results in the “representativeness” of samples from a population. Here, representativeness means that the picture of the population in the sample is unbiased, e.g., the same distribution of factors in the sample as in the population. Random allocation supports the fair (unbiased) treatment comparison, which is difficult – or strictly speaking, not possible to achieve in observational studies.

In 1662, the Flemish physician van Helmont was one of the first to bring the randomization idea into clinical research [14]. He proposed to divide a certain number of diseased patients into two groups by casting lots to evaluate the care of two different persons/treatment strategies. This allocation procedure is more or less what we nowadays call the Random Allocation Rule. Among the most prominent examples, the streptomycin trial in 1948 under the supervision of the Medical Research Council (MRC) was the first, where Bradford Hill’s proposal to favor the use of random allocation instead of alternating sequence [15,16]. So there is already a long tradition of using randomization in clinical trials, based on physicians’ as well as biostatisticians’ recommendations.

Randomization, as described in the ICH E9 [17] guideline, is the process of treatment allocation using an element of chance. It serves as a basis for valid conclusions from the statistical inference. It also tends to produce a similar distribution of prognostic factors in the treatment groups and, in combination with blinding and concealment, it helps to avoid bias in the selection and allocation. This is also supported and reflected in the ICH E8 [18] guideline. The basic reasoning is that by random allocation, it is less predictable which treatment the next patient will receive, and that, taking into account that no inclusion or exclusion criteria are unique, bias could arise from some sort of selection of patients. So we will evaluate whether randomization used in RD clinical trials is capable of mitigating bias. In summary, random allocation should be considered and implemented as the basis of inference and to avoid bias.

Subsequently, we briefly explain different RPs. For comprehensibility, we only consider fixed-sample procedures, which means that no adjustment of the randomization process is made within the ongoing study. For simplification, we introduce the following notation: Let $y_{i}$ be the response of a patient i,1≤i≤N, within the two-arm parallel group randomized clinical trial with total sample size *N*. Denote $N_{E}$ the number of patients randomized to the experimental group (E) and $N_{C}$ the number of patients randomized to the control group (C), respectively, such that $N=N_{E}+N_{C}$. The allocation for each patient *i* is denoted by $T_{i}=1$ if patient *i* is allocated to E and $T_{i}=0$ if patient *i* is allocated to C.

**CR**       Complete randomization: The probability that patient *i* will receive treatment E is 0.5.

**RAR**    Random Allocation Rule: *N*/2 patients are randomized to receive treatment E.

**PBR(*k*)**   Permuted Block Randomization with block size *k*: Patients are randomized in blocks of size k. Implementation of RAR within *N*/*k* Blocks of size *k*.

**BSD(*b*)**   Big Stick design with maximum tolerated imbalance of *b*: The probability that patient *i* will receive treatment E is 0.5. If the imbalance in group sizes reaches *b*, the next patient is deterministically assigned to the treatment with the smaller group size.

**MP(*b*)**    Maximal Procedure with maximum tolerated imbalance of *b*: Uniformly selects from the subset of sequences generated by RAR that never exceed a maximum tolerated imbalance *b* in group sizes during the allocation

**EBC(*p*)**   Efron’s Biased Coin with bias probability *p*: Patients are randomized by flipping a coin with biased probability *p* in favor of the treatment, which is allocated less frequently.

**CHEN(*b*,*p*)**  Chen’s biased coin design with maximum tolerated imbalance of *b* and bias probability of *p*: Patients are randomized by flipping a coin with biased probability *p* in favor of the treatment, which is allocated less frequently. If the imbalance in group sizes reaches *b*, the next patient is deterministically assigned to the treatment with the smaller group size.

What theory tells us is that no RP performs best on all criteria [6,19,20]. (Applied) Scientists usually think skeptically about randomization. The principle of random allocation seems unclear, and randomization is primarly considered as an allocation tool where unequal group size is a major problem. Further recommendations for scientific arguments to select a particular RP do not appear in the ICH guidelines [17,18] nor the CONSORT statement [21].

### 2.2 Evaluation of practice of randomization schemes in rare disease clinical trials

The question now remains to what extent general concepts of randomization are transferable to the RD context. What are the specific requirements of clinical trials in small populations for RPs, and how should available evaluation methods be used under these circumstances? To this end, it is essential to investigate the current status of the use of randomization and blinding in clinical trials on RD. In particular, the freely accessible database Orphanet, which contains extensive data sets on RD and their prevalences, and the Aggregate Analysis of ClinicalTrials.gov (AACT) dataset, which provides information on every trial registered in ClinicalTrials.gov, are suitable for this purpose. A similar approach has already been explored by Hee et al. [22]. We updated this investigation to extend the data extraction about information on randomization and blinding in RD clinical trials, which fell within the following prevalence classes:

i:    1–5/10,000
ii:   1–9/100,000
iii:    1–9/1,000,000
iv:     < 1/1,000,000
v:    unknown

Data linking the Orphanet database and the AACT dataset was extracted in 03/2022 [23] and reanalysed in 03/2025.

#### 2.2.1 Criteria to evaluate randomization schemes.

In the following, we present different criteria for the evaluation of RPs in clinical trials. The underlying concepts are illustrated using PBR(4), and the criteria are classified into indirect and direct criteria.

**Indirect Criteria** Indirect measures are those measures to quantify allocation bias, who describe properties of a particular RP itself, but does not allow to draw a direct conclusion of the test decision. An important example is predictability. Let us consider the randomized and placebo-controlled trial by McDonald et al [24] on the use of Ataluren in 220 patients with nonsense mutation Duchenne muscular dystrophy (ACT DMD) stratified by three factors, each with two levels. ACT DMD is a severe, progressive, and rare X-linked recessive neuromuscular disease, and Ataluren is an orally administered drug designed to mitigate the effects of nonsense mutations. Randomization was carried out using stratified blocks of size 4 (PBR(4)), resulting in 55 blocks. Let us discuss the problem of allocation bias, which is most commonly associated with predictability and the number of correct guesses, such that more suitable patients could be selected for assignment to the preferred treatment [19]. Such bias in clinical trials may also occur when investigators pursue the best intentions [25] through unconscious habits. A more detailed look at the randomization performed in McDonald’s study reveals that each block followed one of the following 6 possible allocation sequences, with “A” corresponding to treatment with Ataluren and “P” corresponding to treatment with placebo:

**Table:** 

AAPP	APAP	APPA	PPAA	PAPA	PAAP

For each of these allocation sequences, the last, or the last two allocations, are deterministic to satisfy the final balance property at the end of the blocks (colored in red). Accordingly, for 60 blocks of size 4, 60–120 of 240 allocations are deterministic and therefore predictable. Considering the same RP with block length 6, it takes 40 blocks of size 6 to obtain the same total sample number of 240 assignments. Here, we obtain 15 different sequences, and the last one, the last two, or the last 3 assignments are deterministic.

**Table:** 

AAAPPP	APAAPP	AAPPPA	AAPAPP	...

As a result, 40–120 treatment assignments are deterministic. Therefore, larger block sizes provide an opportunity for a smaller number of deterministic allocations. Deterministic allocations are a simple indirect measure of allocation bias. The percentage of deterministic allocations is a measure of allocation bias only when the biasing strategy is such that the investigator biases only when the next assignment is deterministic, but not when the investigator biases whenever an imbalance in treatment totals occurs. It is also possible to vary the block length, again randomly, perhaps using a mixture of blocks of size 2, 4, or 6 [26]. Thus, the question is whether it is important to achieve the planned number of assignments to treatment groups and to compensate for patient heterogeneity through stratification. The aim is to achieve a fair treatment comparison without systematic errors. This ultimately leads to a higher credibility of the results and makes them reproducible.

**Direct Criteria**

**Fig 1:**
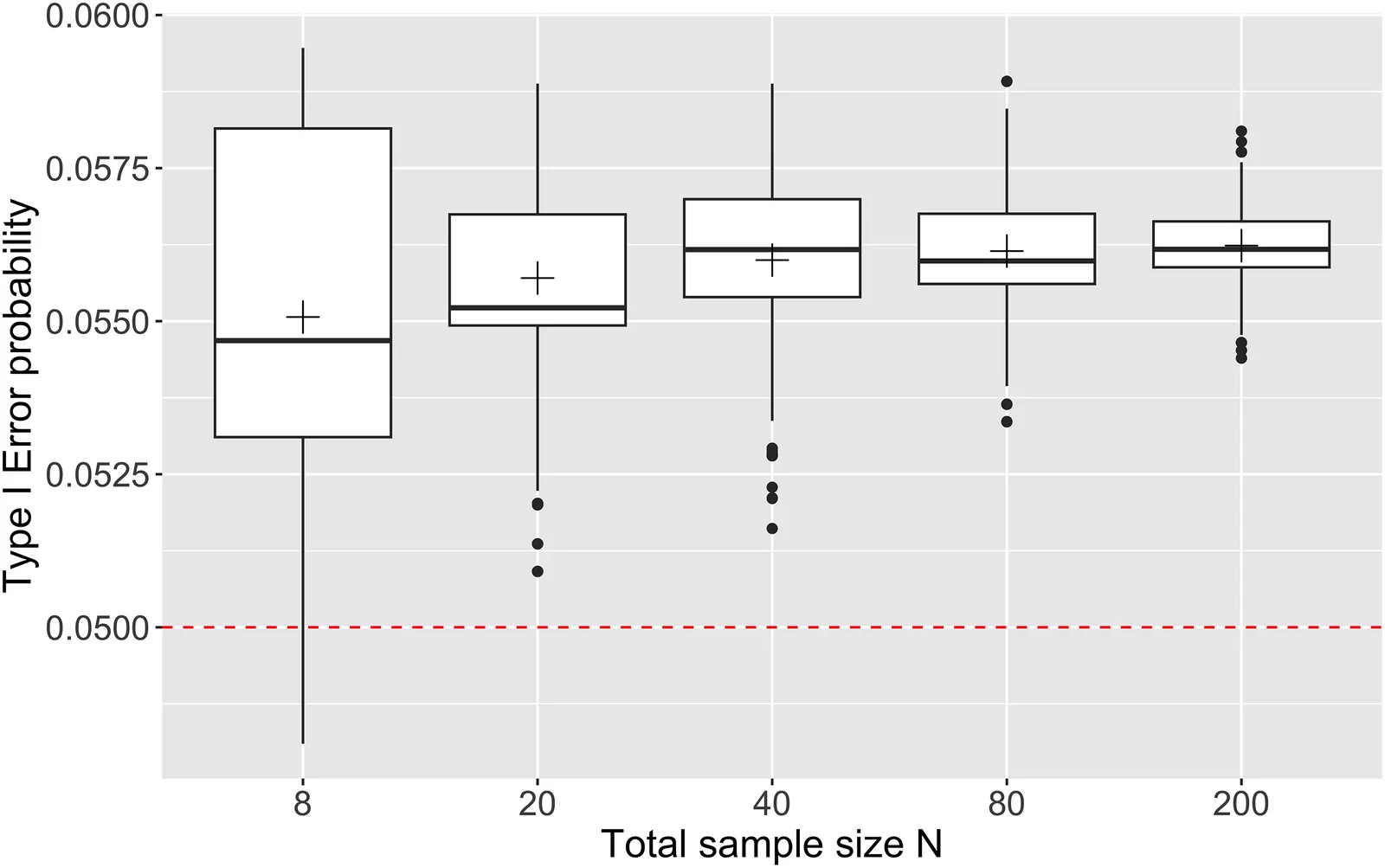
Quantification of Allocation Bias depending on the total sample size for PBR(4) for the Allocation Bias effect of 10% of the treatment effect.

In contrast to the indirect measures, it is also possible to determine and compare direct measures to quantify the bias effect for different RPs, i.e., the mean actual type-1 error probability and the go-no-go criterion. The corresponding approach is given by the ERDO-framework [27], which enables us to select the best bias-mitigating procedure in our clinical setting. The mean actual type-1 error probability is defined as the mean of the actual type-1 error probabilities of the randomization sequences of an RP. In contrast to this, we consider the proportion of actual type-1 error probability *w* generated by the randomization sequences of a particular RP being less than or equal to 0.05 (nominal significance level), denoted by $P_{RP}(w\leq 0.05)$. We call this the go-no-go criterion. While the Blackwell and Hodges model for evaluating a randomization procedure simply counts the number of predictable assignments, our model has been extended to incorporate an assumption regarding the expected allocation effect. The added benefit is that this allows us to quantify the impact on the test decision. An analogy for our method can be found in sample size calculation. Here, assumptions are also incorporated into a model to determine the appropriate number of study participants; we determine the appropriate randomization procedure based on an assumption regarding the expected amount of allocation bias. If we reconsider PBR(4), the impact of the allocation bias effect can be simulated as a function of the sample size and a specific effect size before the trial. The allocation bias effect is assumed to be proportional to the effect size of a trial to be 10%. To balance the effect of increased sample size and decreased effect size we calculated for each of the presented sample sizes (8, 20, 40, 80, and 200) the effect sizes (2.381, 1.325, 0.909, 0.634, and 0.398) that can be detected assuming a power of 80% using a t-test and thus adopt the size of the allocation bias effect accordingly. Fig 1 illustrates this relationship by showing boxplots of the actual type-1 error probabilities for the randomization sequences of PBR(4), including the mean (indicated by “+”) and the median (represented by the horizontal line within each boxplot).

**Fig 2:**
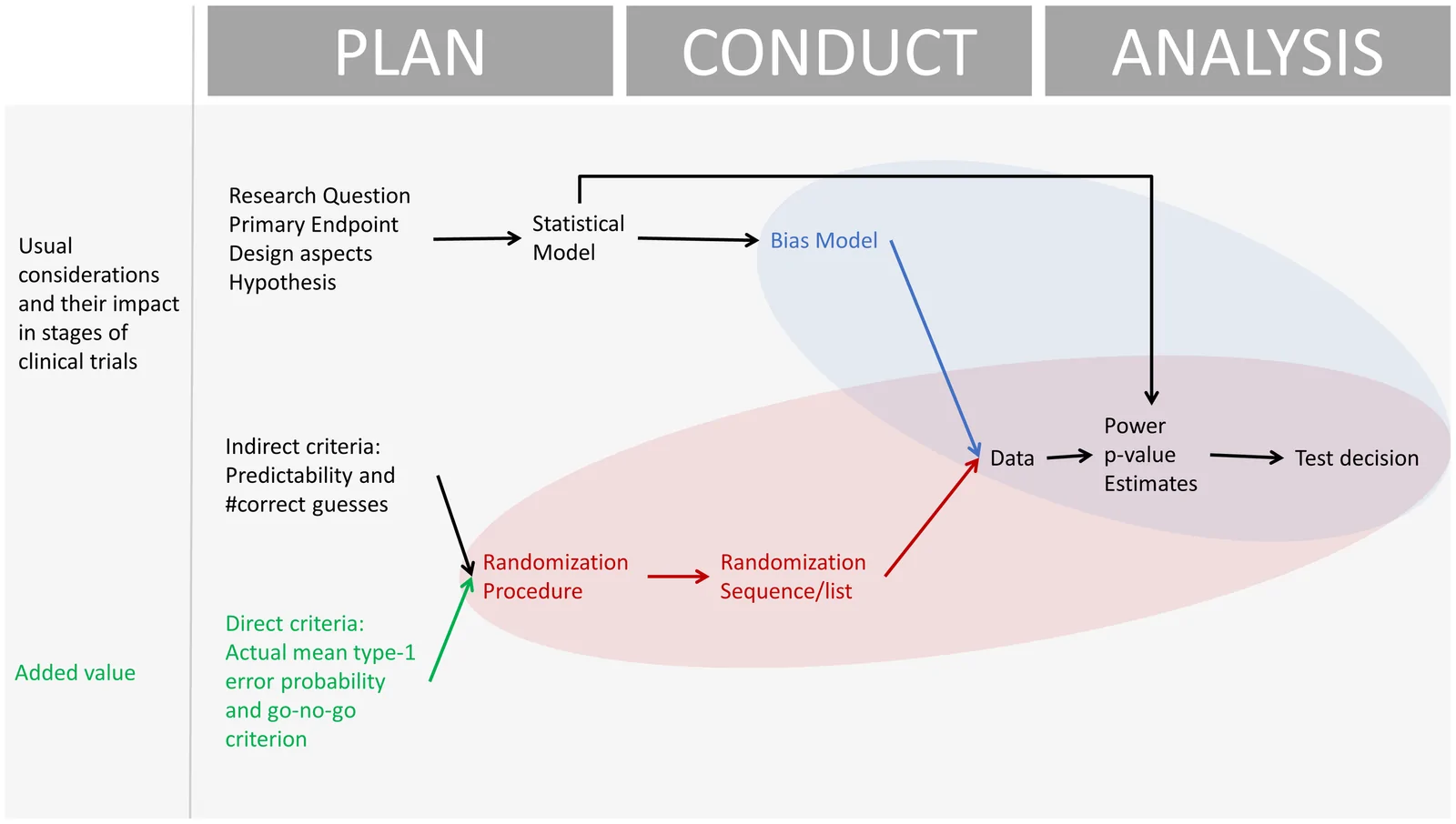
The influence of randomization and bias in different stages of clinical trials.

On the one hand, the actual type-1 error probability varies less with increasing sample size. However, we also see that the actual type-1 error probability itself increases with increasing sample size. This is due to the fact that the proportion of correct guesses remains the same regardless of how many blocks are used in randomization. In our situation, where we test the two-sided null hypothesis $H_{0}:\mu_{E}=\mu_{C}$ of comparing means, the bias in the estimate of the difference in the treatment means remains the same, but the variance of the difference decreases; thus, the type-1 error probability increases. We opted for a two-sided test because the guidelines favor this approach and we want to account for the effects of bias in both directions. However, if one-sided tests are preferred, similar considerations apply. To make allocation bias measurable, one can adopt the following policy [27]:


$$\begin{array}{c} \tau_{i}=\left\{ \begin{array}{cc} \eta & \text{, if }N_{E}(i-1)<N_{C}(i-1)\\ 0 & \text{, if }N_{E}(i-1)=N_{C}(i-1)\\ -\eta & \text{, if }N_{E}(i-1)>N_{C}(i-1), \end{array}\right. \end{array}$$

where η models the allocation bias effect representing the amount of increase or decrease in the expected response. This biasing policy can be described as follows: Whenever there are more patients allocated to one treatment so far ($N_{E}(i-1)<N_{C}(i-1)$ or $N_{E}(i-1)>N_{C}(i-1)$), the recruiter guesses that the next patient is allocated to the other treatment. This biasing policy is based on the assumption that the recruiter prefers treatment E and that higher response values y are associated with better outcomes. Consequently, he or she will unconsciously or intentionally assign the patient with the better response to the preferred treatment E. This relationship is represented by η. Under the null hypothesis $H_{0}:\mu_{E}=\mu_{C}$ and no allocation bias, the 5% level should be adhered to exactly for each randomization sequence when applying the t-test, resulting in $P_{RP}(w\leq 0.05)=1$. Accordingly, we examine the effect of allocation biases associated with a randomization list implemented in the study on the actual type-1 error probability of the test under the miss-classification model. This model under miss-classification is characterized by the fact that we model a allocation bias effect (different from zero), but in the analysis model, we do not consider this effect. This is reflected by the statistical model $y_{i}=\mu_{E}T_{i}+\mu_{C}(1-T_{i})+\tau_{i}+\epsilon_{i}$, where $\epsilon_{i}\sim N(0,\sigma^{2})$. There is a different actual type-1 error probability for each randomization list under the null hypothesis, and a allocation bias effect (different from zero) when the allocation bias effect (different from zero) is not included in the statistical analysis model. We are particularly interested in comparing RPs by the proportion of maintained actual type-1 I error probabilities to support the regulatory decisions. Therefore, we derive by Monte Carlo simulation a distribution of the actual type-1 error probabilities associated with the randomization lists generated by the RP. As described above, there are several ways to evaluate this bias effect. A common approach is to calculate the mean of the actual type-1 error probabilities resulting by the randomization sequences generated by a particular RP. However, this can be misleading because actual type-1 error probabilities can occur below and above 0.05 and thus cancel each other out. This criterion can be referred to as the expected empirical type-1 error probability. Note that the term p-value can be misleading in this situation. The interpretation is as follows: If the mean actual type-1 error probability exceeds the nominal value of 5%, the test is expected to allow more erroneous rejections under the null hypothesis than are normally allowed. The second evaluation criterion is the probability of actual type-1 error probabilities (resulting by the randomization sequences generated by a particular RP) lower than or equal to 0.05. This criterion is called the go-no-go criterion because the cases with an actual type-1 error ≤not 0.05 are consistent with the regulatory requirements for approving a substance, whereas scenarios with an actual type-1 error probability > 0.05 are not. Consequently, higher values of this probability indicate better performance. Altogether, it can be noted that the direct criteria, which can be determined by simulation studies, provide additional value for the allocation of an RP and the analysis of a clinical trial. Fig 2 illustrates this connection.

It is clearly seen that the direct criteria provide extra information compared to the indirect criteria, which are based solely on the RP and its parameters (such as block length). With these simulation studies, it is possible to obtain a guided choice for the best possible RP depending on the study settings and to make the test decision more valid. We aim to implement a model with a measurable bias to quantify the effect on the result of a randomized clinical trial. This is based on the regulatory recognition that bias will take place in every trial [25] and the investigator has to discuss this [17]. In the following, we will present the available methods and give an introduction to the implementation for custom simulation studies focusing exclusively on the presented direct evaluation criteria.

### 2.3 Simulation study with randomizeR

An overview of the methods that have been developed so far can be found in Table 1. A broad range of study designs is already covered, such as two-arm or multi-arm, single- and multi-center, and different types of endpoints. In addition, the methods include several options for modeling different bias effects, such as allocation bias or chronological bias in different types, such as stepwise, linear, and logarithmic.

**Table 1:** Overview of already evaluated study designs with respect to randomization procedures and bias.

	Parallel group design	Endpoint	Hypothesis	Test	Allocation Bias	Chronological Bias
Hilgers et al. [27] (2017)	2-arm single center	continuous	$H_{0}:\mu_{E}=\mu_{C}$	*t*-Test	additive	stepwise, linear, logarithmic
Hilgers et al. [28] (2019)	2-arm multicenter	continuous	$H_{0}:\mu_{E}=\mu_{C}$	stratified *t*-Test	additive	stepwise, linear, logarithmic
Uschner et al. [29] (2018)	multi-arm single center	continuous	$H_{0}:\mu =0$	ANOVA	additive	–
Rückbeil et al. [30] (2017)	2-arm single center	time to event	$H_{0}:\lambda_{E}/\lambda_{C}=1$ $\left(H_{0}:S_{E}=S_{C}\right)$	*F*-/Logrank- Test	multiplicative	stepwise, linear, logarithmic
Schoenen et al. [31] (2024)	2-arm single center	continuous	$H_{0,k}:\mu_{E,k}=\mu_{C,k}$ (k∈{1,...,m},m≥2)	*t*-Test (Šidák, All-or-None)	additive	–
Schoenen et al. [32] (2026)	2-arm multicenter	continuous	$H_{0}:\mu_{E}=\mu_{C}$ $\left(\mu_{E},\mu_{C}\in {\mathbb{R}}^{m},\;m\geq 2\right)$	Wei-Lachin Test (stratified)	additive	–
Bodden et al. [33] (2026)	2-arm group sequential	continuous	$H_{0}:\mu_{E}=\mu_{C}$	*t*-Test	additive	–
Bodden et al. [34] (under Review)	multi-arm platform	continuous	$H_{0}:\mu_{E}=\mu_{C}$	*t*-Test	additive	stepwise, linear, inverted-U, seasonal
Ihl et al. (work ongoing)	2-arm multicenter	binary	$H_{0}:\pi_{E}=\pi_{C}$	Fisher-Test Boshloo-Test	multiplicative	–

In our simulation study, we will demonstrate the easy implementation using the randomizeR package [12] and present the results obtained from the code. To illustrate the distinction between our two primary criteria, the mean actual type-1 error probability and the go-no-go criterion P(w≤0.05), we provide a detailed numerical example in the supplementary material S1 Appendix. Using a small sample size (*N* = 4), this example demonstrates how these criteria are calculated and how they characterize the robustness of a randomization procedure against allocation bias. While this minimal example serves to clarify the underlying logic, we now transition to more realistic, larger-scale settings. We will cover multiple published methods presented in Table 1 and show short code examples as well as results. To use the randomizeR package under R, we recommend using R Studio [35]. After installing the tool, you can download and activate the randomizeR package using the following input in the console of RStudio.

**Listing 1.** Installing randomizeR

*#Install randomizeR package*

**install.packages**(’randomizeR’)

*#Loading library*

**library**(’randomizeR’)

Alternatively, the package can be downloaded from the repository CRAN and installed manually in RStudio [36]. First, we define the general settings. Here, we assume a sample size (N) of 32. As mentioned in section 2.1, we then choose the parameters for the various RPs. We choose a block length (k) of 4, resulting in PBR(4). Furthermore, we choose the maximum tolerable imbalance (b) as 4 and the biased coin probability (p) as 2/3, resulting in BSD(4), MP(4), EBC(2/3), and CHEN(4,2/3). As PBR(4) results in a maximum tolerable imbalance of 2, we additionally include BSD(2) and MP(2) in our investigation. We run all simulations with 16,000 simulation repetitions, i.e., 16,000 randomization sequences, to ensure sufficient precision in estimating the type-1 error rate. Following [19], we can estimate a type-1 error probability as large as 0.04 with a relative error of 10% and a probability of 0.99. For continuous endpoints, we set the expected values under the null hypothesis to 0 (e.g., $\mu_{A}$=0) and the standard deviation to 1 (e.g., $\sigma_{A}$=1). For the design of a 2-arm trial (section 3.2.1, section 3.2.2), we obtain an endpoint defined for 2 groups (A, B) corresponding to an experimental treatment and a standard treatment. For the design of a multi-arm trial (section 3.2.3), we choose 4 arms and accordingly define an endpoint for 4 groups (A, B, C, D). We evaluate the scenarios using the statistical procedures mentioned in Table 1 column “Test”.

**Listing 2.** Global settings for continuous normal endpoints

*#### Defining general settings ####*

N < - 32

k < - 4

b < - 4

p < - 2/3

r < - 16000

seed < - 3003

*# Expectation of groups under null hypothesis*

muA < - muB < - muC < - muD < - 0

*# Standard deviation of groups under null hypothesis*

sigmaA < - sigmaB < - sigmaC < - sigmaD < - 1

*# Normal continuous endpoint for 2-Arm Trials*

endp2arm <- normEndp(mu = c(muA, muB),

         sigma = c(sigmaA, sigmaB))

*# Normal continuous endpoint for 4-Arm Trials*

endp4arm <- normEndp(mu = c(muA, muB, muC, muD),

         sigma = c(sigmaA, sigmaB, sigmaC, sigmaD))

## 3 Results

### 3.1 Randomized rare disease clinical trials in practice

Linking the Orphanet database and the AACT dataset [23], we found that a total of 8802 studies were available at the time of analysis that were conducted in the European Union or the United States, which listed industry, National Institutes of Health (NIH), United States Federal Reserve System (U.S. Fed.) or others as primary sponsors, and of these 8802 studies, we excluded 3323 from our following evaluation because their study design descriptions contained the term “single group” and therefore could not be considered as comparative clinical trials with reasonable relation to randomization, resulting in 5479 comparative trials. From the results in Table 2, it can be seen that there are many studies in which randomization is unknown or missing. However, it is shown that 47.84% of all presented comparative studies were randomized, which represents the importance of randomization in this field of investigations. Only 11.72% of all presented comparative studies are non-randomized. Considering the degree of blinding in RD clinical trials, it becomes clear that a large proportion (34.31%) of the studies conducted are open-label, i.e., without blinding. This means that no additional strategy is implemented to prevent bias. The blinding types single, double, triple, and quadruple altogether only comprise slightly more than 26% of all studies. For the definition of blinding up to quadruple, we refer to [37]. Due to this lack, it is therefore essential to correctly identify and implement the chosen RPs. On the other hand, there is a clear need to make the tool of randomization accessible to a wider community and to demonstrate easy-to-use methods to improve the quality and validity of studies in RD.

**Table 2:** Details of implemented randomization and masking in rare disease clinical trials in relation to prevalence classes in comparative trials registered Orphanet and ClinicalTrails.gov.

		Prevalence class					
		i	ii	iii	iv	unknown	total
count		1746	1999	1124	594	16	5479
Randomization, n (%)	unknown	772 (14.09)	764 (13.94)	522 (9.53)	123 (2.24)	11 (0.20)	2192 (40.01)
	missing	9 (0.16)	14 (0.26)	1 (0.02)	0 (0.00)	0 (0.00)	24 (0.44)
	non-randomized	190 (3.47)	232 (4.23)	139 (2.54)	79 (1.44)	2 (0.04)	642 (11.72)
	randomized	755 (14.14)	989 (18.05)	462 (8.43)	392 (7.15)	3 (0.05)	2621 (47.84)
Blinding, n (%)	unknown	743 (13.56)	758 (13.83)	494 (9.02)	133 (2.43)	11 (0.20)	2139 (39.04)
	Opel Label	579 (10.57)	638 (11.64)	412 (7.52)	250 (4.56)	1 (0.02)	1880 (34.31)
	Single	58 (1.06)	102 (1.86)	42 (0.77)	57 (1.04)	0 (0.00)	259 (4.73)
	Double	115 (2.10)	168 (3.07)	56 (1.02)	61 (1.11)	3 (0.05)	403 (7.36)
	Triple	86 (1.57)	85 (1.55)	35 (0.64)	26 (0.47)	1 (0.02)	233 (4.25)
	Quadruple	165 (3.01)	248 (4.53)	85 (1.55)	67 (1.22)	0 (0.00)	565 (10.31)

### 3.2 Simulation results

#### 3.2.1 ERDO: 2-Arm parallel group design in single center, continuous normal endpoint.

Different randomization sequences are generated for CR, RAR, PBR(4), BSD(4), BSD(2), MP(4), MP(2), EBC(2/3), CHEN(4,2/3).

**Listing 3.** Generating the randomization sequences for different RPs

*#### Generating randomization sequences ####*

crSeq <- genSeq(crPar(N),r=r,seed=seed)

rarSeq <- genSeq(rarPar(N),r=r,seed=seed)

pbrSeq <- genSeq(pbrPar(rep(4,N/4)),r=r,seed=seed)

bsdSeq <- genSeq(bsdPar(N,b),r=r,seed=seed)

bsdSeq2 <- genSeq(bsdPar(N,b/2),r=r,seed=seed)

mpSeq <- genSeq(mpPar(N,b),r=r,seed=seed)

mpSeq2 <- genSeq(mpPar(N,b/2),r=r,seed=seed)

ebcSeq <- genSeq(ebcPar(N,p),r=r,seed=seed)

chenSeq <- genSeq(chenPar(N,b,p),r=r,seed=seed)

We start with a consideration about a two-arm parallel-group design with a continuous endpoint. The simulation is conducted under the (two-sided) null hypothesis of no true treatment effect $H_{0}:\mu_{A}=\mu_{B}$. Nevertheless, we investigate a nominal treatment effect of 1.024, which corresponds to the effect size used in the sample size calculation to achieve 80% power at a two-sided significance level of 5%. For simplicity, we only model a allocation bias effect, which is assumed to be 10% of the nominal treatment effect. Other bias effects, like chronological bias, can be implemented analogously.

**Listing 4.** Setting for treatment effect and allocation bias

d_ERDO <- 1.024

sBiasERDO <- selBias("CS", eta=d_ERDO/10, method="exact")

We use CR as an example to illustrate the implementation and assessment in R. As described before, we account for two possible evaluation criteria, i.e., the mean actual type-1 error probability and the go-no-go criterion. Note that the assessment of all other RPs can be implemented analogously, referring to the respective generated set of randomization sequences, and the entire R-Code can be found in the supplementary material S1 File.

**Listing 5.** Assessment of the evaluation criteria for CR

crAssess_ERDO <- assess(crSeq,sBiasERDO,endp=endp2arm)

crAssess_ERDO  < - crAssess_ERDO$D

crAssess_ERDO  < - c(“CR,”round(mean(crAssess_ERDO[,3]),

         digits=3),round(sum(crAssess_ERDO[,3]<=0.05)

         /length(crAssess_ERDO[,3]),digits=3))

Using the settings described above and the customized code for the respective RPs we receive the results displayed in Table 3. It can be seen that the mean actual type-1 error is slightly increased for all RPs except for CR and BSD(4), where the 5% level can be maintained. Comparing this with the results of the go-no-go criterion, it can be seen that CR and BSD(4) also perform best, as for CR, the value is highest and thus closest to 1, and for BSD(4), the value is high as well. The other RPs only achieve low values in this criterion. We recommend that the overall evaluation of RPs should be based on both criteria. In this case, this means that CR and BSD(4) are the most likely to be recommended, with CR having a mild advantage in the go-no-go criterion. For a 2-arm parallel-group study carried out in one center with the given assumptions, we would therefore recommend one of these two RPs. In the supplementary material S2 Appendix, we included the same investigation for a setting with a nominal significance level of 10% to account for the fact that 5% may be too stringent in RD clinical trials.

**Table 3:** Simulation Results for 2-Arm parallel group Design in one center with continuous normal endpoint.

Randomization Procedure [RP]	Type-I Error [mean]	$P_{RP}(w\leq 0.05)$
CR	0.050	0.464
RAR	0.051	0.139
PBR(4)	0.056	0.000
BSD(4)	0.050	0.372
BSD(2)	0.052	0.086
MP(4)	0.052	0.089
MP(2)	0.054	0.001
EBC(2/3)	0.052	0.108
CHEN(4,2/3)	0.052	0.087

#### 3.2.2 Stratified: 2-Arm parallel group design in two centers, continuous normal endpoint.

For the stratified 2-arm parallel group design, we can use the same general settings. We therefore also use a treatment effect of 1.024 and a allocation bias effect of 10% of the treatment effect.

**Listing 6.** Setting for treatment effect and allocation bias

d_Stra <- 1.024

sBiasStra <- selBias("CS", eta=d_Stra/10, method="exact")

When generating the sequences, we must keep in mind that we want to obtain stratified randomization lists. Therefore, in this case, we generate two lists (one for each of the 2 strata, here centers) covering half the sample size of the total cohort (N/2) and then combine these two sequences. While we here refer to stratification by center, in RD trials, stratification by other relevant factors reflecting population heterogeneity (e.g., age of onset in Friedreich’s ataxia) is also common. The stratification by center is used here as a general term and can be considered synonymous with stratification by any key prognostic factor relevant to the trial population. We present the procedure of evaluation in the following, again using the CR as an example. Once again, please refer to the S1 File for the code implementing the other RPs. We use CR, RAR, PBR(4), BSD(4), BSD(2), MP(4), MP(2), EBC(2/3), CHEN(4,2/3) stratified for two centers.

**Listing 7.** Generating the randomization sequences and parameters for CR

*# Generating sequences*

crSeq1 <- genSeq(crPar(N/2), r = r)

crSeq2 <- genSeq(crPar(N/2), r = r)

crSeq_Stra  < - c(crSeq1, crSeq2)

*# Calculating non-centrality parameters*

crncps  < - genNcps_new(crSeq_Stra,

         c(sBiasStra, sBiasStra),endp2arm)

crdelta  < - crncps[[1]]

crlambda  < - crncps[[2]]

**Listing 8.** Calculation of p-values for CR

*# Calculating p-values*

crp_values  < - sapply(1:length (crdelta), function(i)

          get_p_values_new(delta = crdelta[i], lambda

          = crlambda[i], N = c(N/2,N/2), alpha=0.05))

For the assessment of the RPs, we refer a second time to the two possible evaluation criteria, the mean actual type-1 error probability and the go-no-go criterion.

**Listing 9.** Assessment of the evaluation criteria for CR

crAssess_Strat  < - c(“CR,”round(mean(crp_values),digits = 3),

      round(sum(crp_values<=0.05)/length(crp_values),

      digits = 3))

These settings lead to the results displayed in Table 4.

**Table 4:** Simulation Results for 2-Arm parallel group Design in two centers with continuous normal endpoint.

Randomization Procedure [RP]	Type-I Error [mean]	$P_{RP}(w\leq 0.05)$
CR	0.050	0.329
RAR	0.052	0.007
PBR(4)	0.056	0.000
BSD(4)	0.051	0.254
BSD(2)	0.052	0.084
MP(4)	0.054	0.000
MP(2)	0.054	0.000
EBC(2/3)	0.052	0.059
CHEN(4,2/3)	0.052	0.051

In Table 4, it can be seen that the mean actual type-1 error probability is slightly increased for all RPs except for CR. BSD(4) shows the slightest inflation of the mean actual type-1 error probability compared to the other procedures. Comparing this with the results of the go-no-go criterion, it can be seen that again CR performs best, followed by BSD(4), as here the values are highest and thus closest to 1. The other RPs only achieve very low values in this criterion. In this case, again, CR and BSD(4) are the most likely to be recommended, with CR having a mild advantage in mean actual type-1 error probability and the go-no-go criterion. For a 2-arm parallel-group study carried out in two centers with the given assumptions, we would therefore recommend one of these two RPs.

#### 3.2.3 Multi-Arm: 4-Arm parallel group design in single center, continuous normal endpoint.

For the multi-arm parallel group design, we have to adapt the general settings with some additional options. Here, we include 4 trial arms (K) and define the sample size per arm as 8 (m). Furthermore, the effect size in this scenario is 0.6261 (*f*), and we account for a allocation bias effect of 10% of the effect size.

**Listing 10.** Updated general setting for 4 arms and allocation bias

K  < - 4

m  < - 8

f  < - 0.6261

rho  < - 0.1

sBiasMulti <- selBias("CS2", eta=f∗rho, ’sim’)

The generation of sequences and the assessment of the RPs can be implemented in the same way as described before. Here again, we refer to the example of the CR. Additionally, we take a look at RAR and PBR(4) as the other RPs are not reasonably transferable to the multi-arm setting of this design.

**Listing 11.** Generating the randomization sequences for CR

crSeq_Multi <- genSeq(crPar(N, K),r=r,seed=seed)

**Listing 12.** Assessment of the evaluation criteria for CR

cr_result  < - doublyF_values(crSeq_Multi,sBiasMulti,endp4arm)

cr_error < -mean(cr_result$p)

cr_gng  < - (sum(cr_result$p < 0.05))/r

The results, obtained using these settings, are displayed in Table 5.

**Table 5:** Simulation Results for 4-Arm parallel group Design in one center with continuous normal endpoint.

Randomization Procedure [RP]	Type-I Error [mean]	$P_{RP}(w\leq 0.05)$
CR	0.050	0.606
RAR	0.050	0.311
PBR(4)	0.051	0.000

It can be seen that the mean actual type-1 error probability is slightly increased for PBR(4) compared to CR and RAR. The results of the go-no-go criterion underline that CR and RAR perform better, as the values are higher. In this scenario, the advantage of CR is moderate as almost twice as many sequences result in non-inflated actual type-1 error probabilities. For a 4-arm parallel-group study carried out in one center with the given assumptions, we would therefore recommend CR.

#### 3.2.4 Time to Event: 2-Arm parallel group design in single center, time to event endpoint.

For the time to event setting, we need to update our general setting with respect to specific parameters of exponential outcomes. We define a censoring time of 3 (for example, months) and an accrual time of 1. The censoring rate is 0.0588, and the expected Hazard Ratio is 0.284 for the hazard rates of 0.88 in the control group and 0.25 in the experimental group. The treatment effect in this setting is defined as the logarithm of the Hazard Ratio. Again, we account for a allocation bias effect of 10% of the effect size.

**Listing 13.** Updated general setting for exponential endpint and allocation bias

cenTime  < - 3

accrualTime  < - 1

cenRate  < - 0.0588

HR  < - 0.284

lambdaC  < - 0.88

lambdaE  < - 0.25

treat.effect  < - log(HR)

endp2armExp <- expEndp(lambda = c(lambdaC,lambdaC),

       accrualTime=accrualTime, cenRate=cenRate,

       cenTime = cenTime)

sBiasTTE  < - selBias(“CS,” -treat.effect/10, “exact”)

For the generation of sequences, we refer to the 2-arm parallel group design for one center, as these are obtained in the same way. The assessment of the RPs can be implemented in the following way. We use CR, RAR, PBR(4), BSD(4), BSD(2), MP(4), MP(2), EBC(2/3), CHEN(4,2/3).

**Listing 14.** Assessment of the evaluation criteria for CR

crAssess_TTE <- assess(crSeq,sBiasTTE,endp=endp2armExp)

crAssess_TTE  < - crAssess_TTE$D

crAssess_TTE  < - c(“CR,”round(mean(crAssess_TTE[,3]),

        digits=3),round(sum(crAssess_TTE[,3]<=0.05)/

        length(crAssess_TTE[,3]),digits = 3))

The results, generated from these settings, are displayed in Table 6.

**Table 6:** Simulation Results for 2-Arm parallel group Design in one center with time to event endpoint.

Randomization Procedure [RP]	Type-I Error [mean]	$P_{RP}(w\leq 0.05)$
CR	0.051	0.000
RAR	0.052	0.000
PBR(4)	0.059	0.000
BSD(4)	0.051	0.000
BSD(2)	0.051	0.000
MP(4)	0.053	0.000
MP(2)	0.052	0.000
EBC(2/3)	0.054	0.000
CHEN(4,2/3)	0.054	0.000

It can be seen that the mean actual type-1 error probability is increased for every RP. The slightest inflations of 0.051 are achieved for CR, BSD(4), and BSD(2), followed by RAR and MP(2). Looking at the results of the go-no-go criterion, we can see that we obtained 0 for all RPs. With this, we see that the go-no-go criterion does not give additional value to the choice for the best RP. For a 2-arm parallel-group study with a time-to-event endpoint carried out in one center with the given assumptions, we would therefore recommend CR, BSD(4), or BSD(2) based on the results of the mean actual type-1 error probability.

## 4 Conclusion

In the preceding sections, we have highlighted the importance of randomization in the context of clinical trials, particularly in RD. Please note that a goal of randomization is to balance covariables, by means of balancing both observable and unobservable patient characteristics between treatment groups and obtain a marginal treatment effect that is not confounded by other factors. Although this can be generally achieved in large clinical trials, balance covariables may not be achieved in RD trials with a limited sample size. We outlined which biases are particularly important in RD and where the specific challenges lie in avoiding them. In particular, the association of allocation bias and randomization to reduce and quantify those biases is an important aspect. We considered indirect criteria for comparing RPs, such as predictability or the number of correct guesses. A number of investigations addressing the evaluation of RPs under bias already exist (see, for example [19,38]). These approaches rely on slightly different assumptions regarding the biasing policy (where η=1 is assumed) and employ the indirect evaluation criteria as the direct relation to the treatment effect is not given. In contrast we opted for a direct criterion, i.e., mean actual type-1 error probability and the go-no-go criterion, with a main advantage that randomization is directly linked to the underlying trial design and the influence of bias on the test decision for the treatment effect is quantifiable. With this, an objectifiable assessment of the impact of bias is possible, and a basis for the final analysis using the respective statistical test under the balancing of covariates is provided. Through our examples, our recommendation becomes clear that the evaluation of RPs and a comparison of RPs among each other should consider both of the presented criteria. Considering the mean actual type-1 error probability separately could be misleading and may result in different decisions. The presented methods can also be extended to other scenarios. Among others, extensions for designs with multiple endpoints, group sequential designs, unbalanced group sizes, and for binary endpoints are in progress. Although complete randomization (CR) performed best in the scenarios, it is not universally preferable in practice. While each patient has a 50% probability of being assigned to one group in CR in a two-arm trial, the final balance is not guaranteed, which is a particular concern of investigators in RD trials and often discourages its use. Alternative RPs can mitigate this risk and may therefore be preferable in specific study contexts. Moreover, RPs can be assessed using many different criteria, yet their practical implications and consistent application remain unclear, including in regulatory settings. The majority of RD clinical trials are unblinded, and caution must be given. PBR does, in general, not mitigate allocation bias. However, although some authors are very strict with arguing against PBR, one should bear in mind that in an ideal double blind trial and with some risk for chronological bias [39], PBR shows some advantages. However, we would favor an argumented scientific evaluation with the specific assumptions in the planning phase of a trial. A larger block size can mitigate the drawbacks of PBR. It should be noted that RAR is essentially PBR with the maximum block size. To be able to give such a justification, we developed the open-access R-package randomizeR within the IDeAl funding [40] to support researchers in optimizing the design of RD clinical trials with respect to bias mitigation and thus increase the level of evidence. This concept extends the general design considerations to be specified [41], similar to the essential sample size calculation. Also, more specific study designs, e.g., cross-over designs, provide a further field of investigation, as the process of randomization has specific requirements in these settings. It should be noted that in this framework, the bias effect is simply guessed during evaluation. It is therefore necessary to assume the size of the bias effect in the study planning stage to be able to carry out the evaluation and choice of RPs in a meaningful way. As part of the EPISTOP-IDeAl project of the European Joint Programme on Rare Diseases, evaluations were conducted to estimate the bias effect in existing studies by reanalyzing the study results, adjusting for the underlying bias in order to gain further insights [42]. This approach creates an opportunity to adjust for bias outside of the planning phase, for example, if problems arise during the conducting phase of the study that were not previously foreseeable. Especially in the field of RD, this can occur because of ethical restrictions.

In conclusion, randomization is and will be a key methodology of RD clinical trials. Quantification of the potential effect of allocation bias on the test decision using the ERDO template supported by randomizeR should be a common approach when planning a trial.

## Supporting information

S1 File Randomize R Script.R. (R)

S1 Appendix Minimal Example. (PDF)

S2 Appendix Additional Analysis. (PDF)
