# Supplementary material for: Does randomization matter in rare disease clinical trials
Source: PLoS One. 2026 Sep 2;21(9):e0339427. doi: 10.1371/journal.pone.0339427 (PMC13537556; doi:10.1371/journal.pone.0339427)
Supplement: Supplementary file 1 [file pone.0339427.s002.pdf]

S1 Appendix. Minimal Example

Illustration of the two evaluation criteria mean actual type-1 error probability and go-no-go-criterion

We consider two-arm parallel-group design with a continuous endpoint with the following specifications:

- $N = 4$ (Total Sample Size)
- $\mu_A = \mu_B = 0$ (Expected responses under null hypothesis)
- $\sigma_A = \sigma_B = 0$ (Standard deviation under null hypothesis)
- $d = 5.653$ (Effect size to achieve 80% Power at two-sided significance level of 5%)
- $\eta = \frac{d}{10}$ (modelled Allocation Bias)

We use RAR as an example to illustrate the generation of randomization sequences and calculation of the two evaluation criteria, i.e., the mean actual type-1 error probability and the go-no-go criterion. This can be implemented using randomizeR like described in Listing 1.

Listing 1. Minimal example

```
install.packages('randomizeR')
library('randomizeR')
#### Defining general settings ####
N <- 4                    # Total sample size

# Expectation of groups under null hypothesis
muA <- muB <- 0

# Standard deviation of groups under null hypothesis
sigmaA <- sigmaB <- 1

# Normal continuous endpoint for 2-Arm Trials
endp2arm <- normEndp(mu = c(muA, muB), sigma = c(sigmaA, sigmaB))

#### ERDO ####
d_ERDO <- 5.653

# Allocation bias
sBiasERDO <- selBias("CS", eta = d_ERDO/10, method = "exact")
```

The simulation of all possible 6 Randomization Sequences (RS) of the Randomization Procedure (RP) Random Allocation Rule (RAR) can be obtained and assessed with the code presented in Listing 2.

Listing 2. Assessment of the evaluation criteria for RAR

```
#### Generating randomization sequences for RAR ####
rarSeq  <- getAllSeq(rarPar(N))
rarAssess_ERDO <- assess(rarSeq,sBiasERDO,endp=endp2arm)
rarAssess_ERDO <- rarAssess_ERDO$D
```

This results in the actual type-1 error probabilities ($w(RS_i)$) for each of randomization sequences $RS_i,\ i = 1, \ldots, 6$ displayed in table 1.

i	RS_i	Probability	$w(RS_i)$
1	BBAA	0.166666667	0.04996349
2	BABA	0.166666667	0.06468513
3	ABBA	0.166666667	0.05597209
4	BAAB	0.166666667	0.05597209
5	ABAB	0.166666667	0.06468513
6	AABB	0.166666667	0.04996349

Table 1. Numerical example of sequence-specific actual type-1 error probabilities (w) for $N = 4$ under a allocation bias model for RAR.

The mean actual type-1 error probability for RAR can be calculated as

$$\frac{1}{6}\sum_{i=1}^{6} w(RS_i) = 0.057.$$

The go-no-go criterion $P(w \leq 0.05)$ for RAR can be calculated as

$$\frac{1}{6}\sum_{i=1}^{6} \mathbb{1}_{(w(RS_i)\leq 0.05)} = \frac{2}{6} \approx 0.333.$$

This calculation can be implemented in randomizeR using Listing 3.

Listing 3. Calculation of evaluation metrics

```
Eval_ERDO <- c("RAR","5%",round(mean(rarAssess_ERDO[,3]),
               digits=3),round(sum(rarAssess_ERDO[,3]<=0.05)/
               length(rarAssess_ERDO[,3]),digits=3))
names(Eval_ERDO) <- c("Randomization Procedure",
               "Significance Level","Type I Error [mean]",
               "P(w<=0.05)")
```

It can be seen that the mean actual type-1 error of 0.057 is slightly increased for RAR. Comparing this with the results of the go-no-go criterion, it can be seen that 2 out of 6 RS maintain the nominal level of 0.05.
