# Supplementary material for: Does randomization matter in rare disease clinical trials
Source: PLoS One. 2026 Sep 2;21(9):e0339427. doi: 10.1371/journal.pone.0339427 (PMC13537556; doi:10.1371/journal.pone.0339427)
Supplement: Supplementary file 2 [file pone.0339427.s003.pdf]

S2 Appendix. Additional Analysis

ERDO: 2-Arm parallel group Design in single center, continuous normal endpoint with nominal significance level of 10%

We consider a two-arm parallel-group design with a continuous endpoint. The simulation is conducted under the null hypothesis of no true treatment effect $H_0 : \mu_A = \mu_B$. Nevertheless, we investigate a nominal treatment effect of 0.9, which corresponds to the effect size used in the sample size calculation to achieve 80% power at a two-sided significance level of 10%. For simplicity, we only model a allocation bias effect, which is assumed to be 10% of the nominal treatment effect. Other bias effects, like chronological bias, can be implemented analogously.

Listing 1. Setting for treatment effect and allocation bias

```
d_ERDO2 <- 0.9
sBiasERDO2 <- selBias("CS", eta=d_ERDO2/10, method="exact")
```

We use CR as an example to illustrate the implementation and assessment in R. As described before, we account for two possible evaluation criteria, i.e., the mean actual type-1 error probability and the go-no-go criterion. Note that the assessment of all other RPs can be implemented analogously, referring to the respective generated set of randomization sequences, and the entire R-Code can be found in the supplementary material S1 File.

Listing 2. Assessment of the evaluation criteria for CR

```
crAssess_ERDO10 <- assess(crSeq,sBiasERDO2,endp=endp2arm)
crAssess_ERDO10 <- crAssess_ERDO10$D
crAssess_ERDO10 <- c("CR",round(mean(crAssess_ERDO10[,3]),
                   digits=3),round(sum(crAssess_ERDO10[,3]<=0.05)
                   /length(crAssess_ERDO10[,3]),digits=3))
```

Using the settings described above and the customized code for the respective RPs we receive the results displayed in table 1. It can be seen that the mean actual type-1 error

Randomization Procedure [RP]	Type-I Error [mean]	$P_{RP}(w \leq 0.05)$
CR	0.100	0.522
RAR	0.101	0.128
PBR(4)	0.107	0.000
BSD(4)	0.100	0.385
BSD(2)	0.102	0.074
MP(4)	0.102	0.070
MP(2)	0.104	0.001
EBC(2/3)	0.102	0.107
CHEN(4,2/3)	0.103	0.083

Table 1. Simulation Results for 2-Arm parallel group Design in one center with continuous normal endpoint for a nominal significance level of 10%.

is slightly increased for all RPs except for CR and BSD(4), where the 10% level can be maintained. Comparing this with the results of the go-no-go criterion, it can be seen that CR and BSD(4) also perform best, as for CR, the value is highest and thus closest to 1, and for BSD(4), the value is high as well. The other RPs only achieve low values in this criterion. We recommend that the overall evaluation of RPs should be based on both criteria. In this case, this means that CR and BSD(4) are the most likely to be

recommended, with CR having a mild advantage in the go-no-go criterion. For a 2-arm parallel-group study carried out in one center with the given assumptions, we would therefore recommend one of these two RPs.
